# Combination therapies with ponatinib and asciminib in a preclinical model of chronic myeloid leukemia blast crisis with compound mutations

**DOI:** 10.1038/s41375-024-02248-0

**Published:** 2024-04-13

**Authors:** Nikola Curik, Adam Laznicka, Vaclava Polivkova, Jitka Krizkova, Eva Pokorna, Pavel Semerak, Pavla Suchankova, Pavel Burda, Andreas Hochhaus, Katerina Machova Polakova

**Affiliations:** 1 Institute of Hematology and Blood Transfusion, Prague, Czech Republic; 2 Institute of Pathological Physiology, First Faculty of Medicine, Charles University, Prague, Czech Republic; 3 Second Faculty of Medicine, Charles University, Prague, Czech Republic; 4 Abteilung Hämatologie/Onkologie, Klinik für Innere Medizin II, University of Jena, Jena, Germany

**Keywords:** Cancer models, Chronic myeloid leukaemia, Preclinical research

## To the Editor:

Despite significant progress in chronic myeloid leukemia (CML) therapy over recent decades, the blast crisis (BC) remains a therapeutic challenge. This advanced disease stage exhibits clonal, genomic, and molecular heterogeneity, often featuring additional chromosomal aberrations and mutations in the BCR::ABL1 kinase domain and/or other leukemia-related genes. Consequently, the efficacy of tyrosine kinase inhibitors (TKIs) is limited [1, 2]. A minority of CML-BC patients achieve complete cytogenetic or hematological responses with TKI monotherapy before transplantation [3, 4]. Moreover, these responses are typically short-lived, resulting in a median overall survival of 6-11 months from diagnosis, with a worse prognosis for myeloid BC [5]. The introduction of 2nd and 3rd generation TKIs in combination with intensive chemotherapy regimens or demethylating drugs has modestly improved responses and survival rates [6, 7]. However, patients failing multiple TKIs, including 3rd generation ponatinib, face unsatisfactory outcomes with limited available treatment options [5]. Recently, the “Specifically Targeting the ABL Myristoyl Pocket” (STAMP) inhibitor asciminib demonstrated effectiveness in heavily pretreated CML patients, especially those in the chronic phase, including those with the BCR::ABL1 T315I mutation or those who experienced ponatinib failure [8]. Presumably, it may also demonstrate efficacy in patients who progressed to blast crisis while on ponatinib [9]. Notably, a robust synergistic antileukemic effect was observed with the combination of ponatinib and asciminib in CML-BC cell lines and CML-BC stem cells carrying the BCR::ABL1 T315I mutation [10]. Moreover, this effect was also evident in murine Ba/F3 cells expressing T315I-inclusive BCR::ABL1 compound mutations, both in vitro and in vivo [11, 12]. In addition to asciminib, a retrospective study assessed the impact of combining ponatinib with the BCL2 inhibitor venetoclax, yielding promising results in heavily pretreated CML-BC patients [13].

**Fig. 1:**
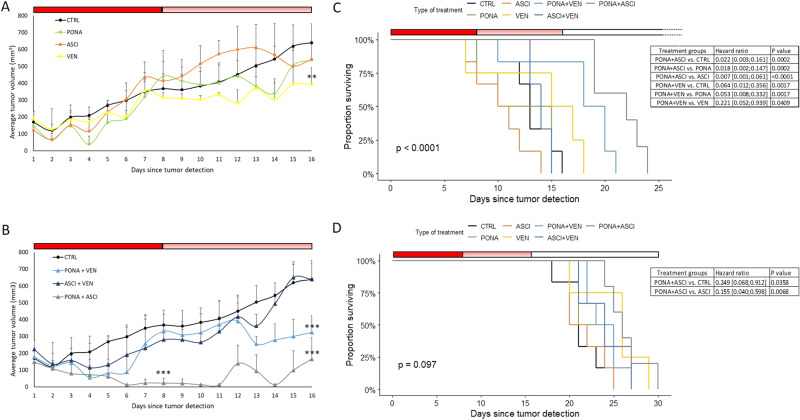
The impact of drug treatment on tumor growth and survival in vivo. The average tumor volume was calculated by measurement of tumors growth in groups of mice according to solo (**A**) or combination (**B**) treatment since the day of tumor detection. The full red line indicates the daily dosing period, the paler red line indicates the period of intermittent regime, the white line indicates the period without treatment. The levels of significance are indicated: **p < 0.01; ***p < 0.001. Error bars represent standard deviations. Event-free (**C**) and overall (**D**) survival of mice according to the treatment groups. Differences in groups were evaluated by type 2 ANOVA F TEST. Significant and relevant differences are displayed in tables. CTRL control group without treatment, PONA mice treated with ponatinib, ASCI mice treated with asciminib, VEN mice treated with venetoclax, PONA + VEN mice treated with asciminib and venetoclax; ASCI + VEN mice treated with asciminib and venetoclax, PONA + ASCI mice treated with ponatinib and asciminib.

In this study, a murine model of polyclonal aggressive myeloid CML-BC was established using imatinib/ponatinib cross-resistant (IMP-R) clones to evaluate the efficacy of ponatinib, asciminib, venetoclax, and their combinations in vivo within a CDX (cell line-derived xenograft) context. Five IMP-R clones of KCL-22 cells were derived from an imatinib-resistant parental clone carrying the BCR::ABL1 T315I mutation [14]. IMP-R clones were established by single-cell FACS sorting imatinib-resistant clone into the 96-well plates containing 0.004 nM ponatinib. Growing clones were then transferred to the medium with an order higher concentration of ponatinib (i.e. 0.04 nM). The process was repeated with stepwise increase (one order per month up to 10 nM; detailed in Supplementary methods). These clones underwent characterization to identify somatic mutations in the *BCR::ABL1* kinase domain and other cancer-related genes (Supplementary methods and Supplementary Table 1). Alongside a common mutation background, clone-specific *BCR::ABL1* compound mutations and mutations in other leukemia-related genes were identified, including Clone 1P2 (BCR::ABL1-T315I + H396R + I418T), Clone 1P3 (BCR::ABL1-T315I + E255V; IKZF1-E383G), Clone 1P4 (BCR::ABL1-T315I + H396R), Clone 1P5 (BCR::ABL1-T315I + E250G; SETD1B-G1963fs), and Clone 1P6 (BCR::ABL1-T315I + Y253H; ZRSR2-R451H). Subsequently, an equal number of cells (1 × 10^6^) from individual IMP-R clones were mixed and subcutaneously xenotransplanted into immunodeficient mice (5 × 10^6^ cells/mouse; *n* = 42). The mice were randomly assigned to 7 groups (*n* = 42; 6 per group) based on the treatment plan: (1) untreated control (CTRL); (2) ponatinib (PONA) 25 mg/kg b.w.; (3) asciminib (ASCI) 30 mg/kg b.w.; (4) venetoclax (VEN) 50 mg/kg b.w.; (5) PONA + VEN; (6) ASCI + VEN; (7) PONA + ASCI (Fig. 1A). Treatment regimens were orally administered once a day from the onset of measurable tumors (5 mm in one dimension) until day 8. Thereafter, the treatment was modified to intermittent dosing due to a weight loss exceeding 10% in venetoclax-combi groups and was discontinued on day 16 due to persistent or increased weight loss in ponatinib-combination groups (Supplementary Fig. S1). Days 8 and 16 were selected as timepoints for evaluating the anti-tumor efficacy of treatment. Among the individual drugs, only venetoclax significantly suppressed tumor growth compared to the control on day 16 (Fig. 1A). Concerning combination regimens, the ponatinib + asciminib treatment exhibited the most effective impact on tumor growth. It significantly suppressed tumor growth on day 8 and reduced tumor volume to the limit of measurability during the daily dosing period, extending until day 11. Moreover, both ponatinib + venetoclax and ponatinib + asciminib combination therapies also significantly suppressed tumor growth measured at the end of intermittent dosing on day 16 (Fig. 1B).

Subsequently, the efficacy of individual drugs and their combinations was assessed through event-free survival (EFS) analysis, defining an event as a tumor volume ≥500 mm³. The treatment regimens had a significant impact on outcomes (*p* < 0.001) (Fig. 1C). PONA + ASCI [HR 0.022; 0.003–0.161; *p* = 0.0002] and PONA + VEN [HR 0.064; 0.012–0.356; *p* = 0.0017] regimens significantly prolonged EFS compared to untreated controls, with median survival times of 22, 19, and 13 days, respectively. In contrast to other regimens, the first event occurred in the PONA + ASCI group only after treatment cessation. Furthermore, both mentioned combinations significantly prolonged EFS compared to the respective solo drug dosing, while neither drug alone provided a survival benefit (Fig. 1C). Overall survival (OS) was also determined, with mice continuously euthanized upon meeting the criteria: tumor diameter ≥20 mm in one direction or skin necrosis. The analysis did not reveal a significant overall impact of treatment regimens on OS, apparently due to the stopping of treatment at day 16 (*p* = 0.097). Nevertheless, the ponatinib + asciminib combination modestly but significantly prolonged OS compared to controls, with median survival times of 26 and 21 days, respectively (Fig. 1D).

**Fig. 2:**
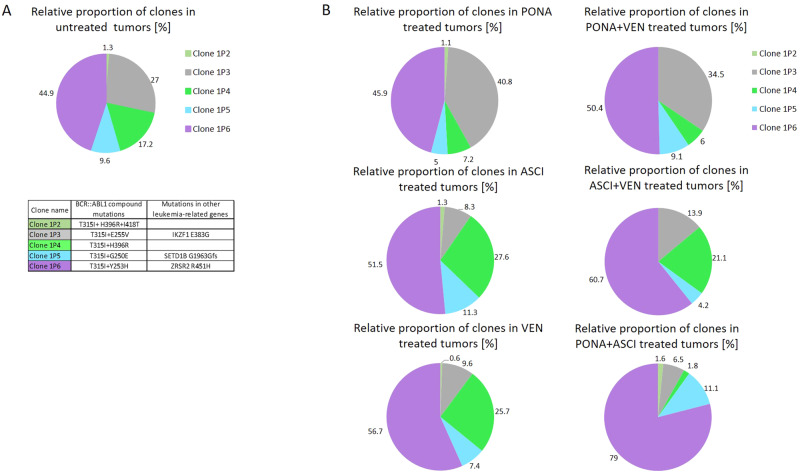
Clonal distribution in tumors. The relative proportion of individual IMP-R clones in untreated tumors (**A**) and tumors subjected to various drug regimens (**B**) is illustrated by charts, with values expressed as percentages of the total. The table provides clone-specific mutations in *BCR::ABL1* and other leukemia-related genes that were acquired during imatinib/ponatinib resistant clone establishment.

After euthanasia, tumors were excised and homogenized using a 70 µm cell strainer. RNA was isolated, and NGS mutational analysis of the *BCR::ABL1* kinase domain was conducted to determine the proportional presence of individual IMP-R clones. Analysis of untreated tumors revealed the dominant presence of Clone 1P3 and Clone 1P6 with compound mutations T315I + E255V and T315I + Y253H, respectively (Fig. 2A). The impact of treatments (8 days of daily dosing + 8 days of intermittent regime) on the vitality of individual clones was assessed by determining the relative clone proportions in tumors of each group (Fig. 2B). The analysis indicated that the relative representation of Clone 1P4 (T315I + H396R) decreased in ponatinib and ponatinib-combination regimens, while the relative proportion of Clone 1P3 (T315I + E255V) decreased in venetoclax and asciminib-inclusive regimens. Clone 1P5 (T315I + E250G) and particularly Clone 1P2 (T315I + H396R + I418T) were relatively weakly represented in all treatment groups, with both venetoclax- combi regimes completely eliminating Clone 1P2. Conversely, Clone 1P6 (T315I + Y253H) was the most represented clone in tumors among all groups, especially in both asciminib-combi regimes, suggesting its overgrowth from residual tumor in ponatinib-asciminib regime following treatment cessation (Fig. 2B). Fold changes (FC) of the clone’s relative proportion relative to untreated controls were determined for ponatinib, asciminib, and combination ponatinib + asciminib treatment and compared to the respective IC_50_ proliferation values of individual clones obtained in vitro (Supplementary Fig. S2). The proportion FC of clones exposed to ponatinib in vivo followed a descending order: Clone 1P3 > Clone 1P6 > Clone 1P2 > Clone 1P5 > Clone 1P4, while the inverse effect on FC was found in the asciminib regime: Clone 1P4 > Clone 1P5 > Clone 1P6 > Clone 1P2 > Clone 1P3. During combinatory ponatinib + asciminib therapy, tumors were unmeasurable during daily drug administration due to the simultaneous activity of both drugs. IC_50_ values for ponatinib and asciminib solidly correlated with FC of clones’ proportions in tumors treated by respective drugs in vivo and were of predictive value. In vitro data also showed synergy in the ponatinib + asciminib combination against IMP-R clones except for Clone 1P2 (T315I + H396R + I418T) (Supplementary Fig. S2B).

In conclusion, despite the highly resistant nature of the mixture of CML myeloblasts, each harboring distinct T315I-inclusive compound mutations, combinatory therapy with asciminib and ponatinib proved potent in suppressing tumor growth in a preclinical animal model to an immeasurable extent. This combination also conferred a significant survival advantage over untreated controls and both drugs used individually. Additionally, the combination of ponatinib and venetoclax demonstrated efficacy in delaying tumor growth, particularly against the growth of the clone with the T315I + H396R + I417T compound mutation. While compound mutations are generally rare in chronic phase CML, their frequency significantly increases in advanced disease, with the therapeutically challenging T315I-inclusive mutations being among the most common [15]. The promising results of this study, consistent with previous findings [10–12], underscore the need for ongoing pre-clinical testing of combination regimens using CML-BC patient-derived xenograft models. While acknowledging the potential significant benefits of salvage therapy for CML-BC patients, it is important to address the issue of toxicity. Although the daily monitoring of animal weight was an only measurable marker of potential toxicity of the therapy, the combination of 30 mg/kg b.w. asciminib and 25 mg/kg b.w. ponatinib did not exhibit exceptional toxicity, as evidenced by the comparison of animal weight loss during the daily dosing period. However, in clinical practice, there may be room for further reduction of the dose specially of ponatinib (asciminib is well tolerable) without compromising efficacy, as suggested previously [11, 16]. Clinical investigations are underway to explore the potential of asciminib in combination with TKIs as a viable option for CML-BC patients following TKI failure (NCT03595917; NCT02081378). These findings contribute to the growing body of evidence supporting the exploration of combination therapies for improved outcomes in CML-BC patients.

## Supplementary information

Supplemental material
