# Supplementary material for: Combination therapies with ponatinib and asciminib in a preclinical model of chronic myeloid leukemia blast crisis with compound mutations
Source: Leukemia. 2024 Apr 13;38(6):1415–8. doi: 10.1038/s41375-024-02248-0 (PMC11147753; doi:10.1038/s41375-024-02248-0)
Supplement: Supplementary file 1 — Supplemental material [file 41375_2024_2248_MOESM1_ESM.pdf]

SUPPLEMENTARY METHODS

CML cell line and establishing imatinib/ponatinib (IMP-R) cross-resistant clones

The KCL-22 cell line (ACC 519) was acquired from a publicly accessible biological resource center (the Leibniz Institute - Deutsche Sammlung von Mikroorganismen und Zellkulturen GmbH/DSMZ, Braunschweig, Germany). Mycoplasma contamination was tested no later than one month after the cells were thawed. Imatinib/ponatinib-resistant clones (IMP-R; n=5) were established from imatinib-resistant Clone 1, described previously [1], by single-cell FACS sorting (BD FACS Aria) into 96-well plates containing 0.004 nM ponatinib, with a stepwise increase (one order of magnitude per month up to 4 nM) up to 10 nM.

Next-generation sequencing (NGS)

The IMP-R clones underwent characterization to identify somatic mutations in the BCR::ABL1 kinase domain and other cancer-related genes. BCR::ABL1 kinase domain amplicon libraries were prepared using the Nextera XT DNA Library Prep Kit (Cat. No. FC-131-1096, Illumina, San Diego, CA, USA). Data processing, error filtering, and mutation calling at significant levels were performed using the NextGene software (Softgenetics, State College, PA, USA) and the in-house bioinformatic tool NextDom. Libraries for the DNA custom NGS panel of 62 genes frequently mutated in hematological malignancies (see Supplementary Table S1) were prepared using the SeqCap EZ HyperCap Workflow (Roche, San Diego, CA, USA) following the manufacturer's instructions. NGS libraries were sequenced on the MiSeq System (Illumina), and data evaluation was conducted using NextGene Software (SoftGenetics). The clinical relevance of the detected variants was assessed in the VarSome open-source database [2].

CDX model

For the establishment of the CDX model, female immunodeficient mice NOD.Cg-PrkdcscidIl2rgtm1Wjl/SzJ (n=42) aged 8-10 weeks were utilized. Number of animals per group (n=6) was calculated based on the estimated effect size of the drug on tumor growth, estimated variability (standard deviations), significance level and Power (0.9) using freeware software Experimental Design Assistant (Group and sample size | NC3Rs EDA). The number of animals was further adjusted in consideration of possible exclusions due to mouse injury during drug application or ineffective xenotransplantation. Mice were randomly and equally allocated to the control and experimental groups. Mice were weighed, and tumor sizes were measured daily with calipers. Euthanasia criteria included: 1) tumor growth to 20 mm in one dimension; 2) visible physical or behavioral discomfort/skin necrosis. The assessment of drug effects on tumors and animals was unblinded. The experimental design was approved by the institutional Animal Care and Use Committee (MSMT-37334/2020-4) and the Ministry of Agriculture of the Czech Republic.

Sensitivity of cells to ponatinib and asciminib *in vitro*, IC_{50} determination

$2x10^5$ cells of IMP-R clones were cultivated in RPMI-1640 medium with asciminib/ponatinib solo in dilution series (0-1000 nM) or with combination of 10 nM ponatinib with asciminib in dilution series (0-1000 nM) for 48 hours. Following cultivation, changes in proliferation for IMP-R clones were measured in tetraplicate using the cell proliferation kit (WST-1 Assay Kit, Cat. No. ab65475, Abcam PLC, Cambridge, UK) with a spectrophotometer Synergy HT (BioTek, Winooski, VT, USA). IC_{50} values were calculated using freeware software AAT Bioquest (https://www.aatbio.com/tools/ic50-calculator).

Statistical analysis

In vivo tumor growth was assessed using the type II ANOVA F test with the Kenward-Roger approximation for the denominators of degrees of freedom. Tumor growth curves among treatment groups were analyzed at a significance level of 95%. Additionally, pairwise comparisons of tumor growth were conducted between treatment groups (SL 95%). Survival probabilities and cumulative incidence rates were estimated using the Kaplan-Meier method. Survival and time-to-event were compared using the log-rank test. The comparison of risk ratios between groups was determined using the Cox proportional risk model at the significance level of 95%.

SUPPLEMENTARY REFERENCES

[1] Curik N, Polivkova V, Burda P, Koblihova J, Laznicka A, Kalina T, et al. Somatic Mutations in Oncogenes Are in Chronic Myeloid Leukemia Acquired De Novo via Deregulated Base-Excision Repair and Alternative Non-Homologous End Joining. Front Oncol. 2021 Sep 20;11:744373

[2] Kopanos C, Tsiolkas V, Kouris A, Chapple CE, Albarca Aguilera M, Meyer R, et al. VarSome: the human genomic variant search engine. Bioinformatics 2019 Jun 1;35:1978-1980

Supplementary Table S1

DNA custom NGS panel of 62 genes frequently mutated in haematological malignancies

Fully covered genes (n=22)		Partialy covered genes (n=40)	
BCOR	full	ABL1	4-10
BCORL1	full	ASXL1	12
CDKN2A	full	ATRX	8-10, 17-31
CEBPA	full	BCL2	2 ,3 both partly
DNMT3A	full	BRAF	15
ETV6	full	CBL	8, 9
EZH1	full	CBLB	9, 10
EZH2	full	CREBBP	24-30
GNB1	full	CRLF2	6
GNB2	full	CSF3R	14-17
IKZF1	full	EP300	24-30
KDM6A	full	FBXW7	9-11
PAX5	full	FLT3	14, 15, 20
PHF6	full	GATA1	2
PRPF8	full	GATA2	2-6
RUNX1	full	IDH1	4
SETD1B	full	IDH2	2, 4
SETD2	full	JAK1	14-16
SF1	full	JAK2	12, 14
SIRT1	full	JAK3	13
UBE2A	full	KIT	2, 8-19
ZRSR2	full	KRAS	2, 3
		MCL1	1, 2, 3 partly
		MLL (KMT2A)	5-11
		MPL	10
		NF1	18
		NOTCH1	26-28, 34
		NPM1	11
		NRAS	2, 3
		PDGFRA	12, 14, 18
		PTEN	5, 7
		PTPN11	3, 13
		SETBP1	4 partly
		SF3B1	12-16
		SMC3	10,13,19,23,25+28
		SRSF2	1
		TET2	3-11
		TP53	2-11
		U2AF1	2, 6
		WT1	4-9

Supplementary figure S1

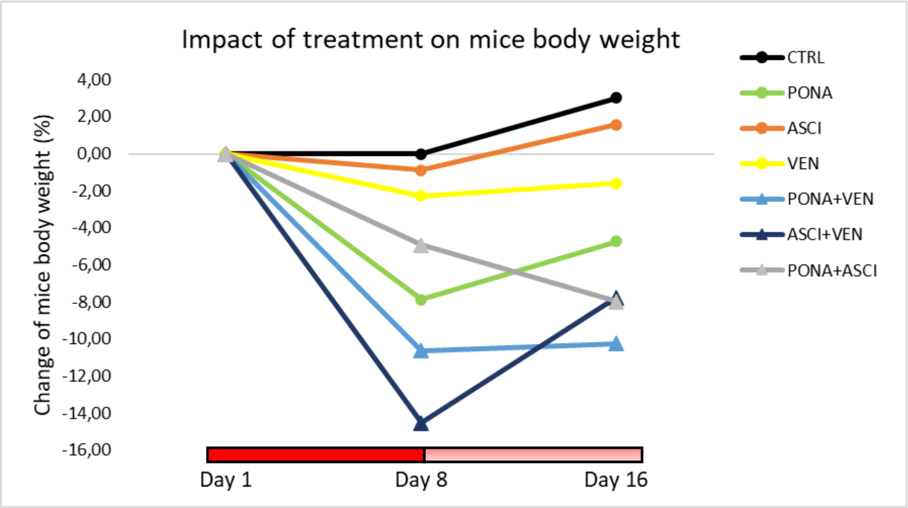

Supplementary Figure S1. **The impact of treatment regimens on animal weight**. The median body weight of mice on days 8 and 16 following tumor detection, expressed as a percentage relative to the initial weight of the animals. CTRL – untreated controls; PONA – ponatinib; ASCI – asciminib; VEN – venetoclax; PONA+ASCI – ponatinib and asciminib; ASCI+VEN – asciminib and venetoclax; PONA+ASCI – ponatinib and asciminib. The full red line indicates the daily dosing period, the paler red line indicates the period of intermittent regime.

Supplementary figure S2

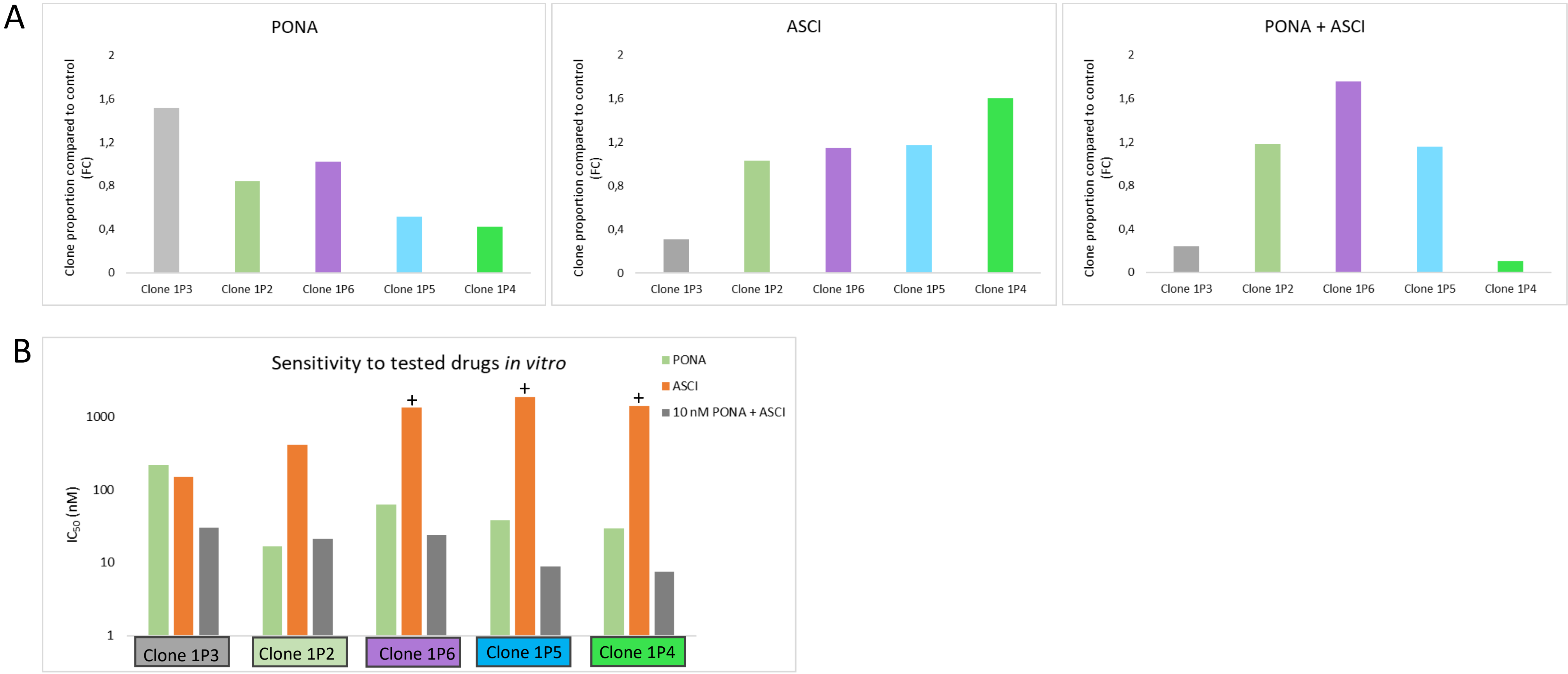

Supplementary Figure S2. Comparing clone growth characteristics *in vivo* and *in vitro*. **(A)** The growth properties of clones under ponatinib (PONA), asciminib (ASCI), and the combination of ponatinib and asciminib (PONA + ASCI) regimens were evaluated as fold changes (FC) in clone proportions within treated tumors relative to untreated controls *in vivo*. Additionally, the same clones were assessed *in vitro* **(B)**, with their growth properties represented by IC_{50} values measured after 48 hours of treatment. + - Measured IC_{50} exceeded the highest applied concentration (1000 nM).
